# A Redundant-Sensing-Based Six-Axis Force/Torque Sensor Enabling Compactness and High Sensitivity

**DOI:** 10.3390/s26030871

**Published:** 2026-01-28

**Authors:** Seung Yeon Lee, Jae Yoon Sim, Dong-Yeop Seok, Yong Bum Kim, Jaeyoon Shim, Uikyum Kim, Hyouk Ryeol Choi

**Affiliations:** 1 Department of Mechanical Engineering, Sungkyunkwan University, Suwon-si 16419, Republic of Korea; sylee6051@skku.edu (S.Y.L.);; 2 AIDIN ROBOTICS Inc., Anyang-si 14055, Republic of Korea; 3 Department of Mechanical Engineering, Ajou University, Suwon-si 16499, Republic of Korea

**Keywords:** capacitive force/torque sensor, compact sensor design, redundant capacitive sensing, reduced coupling error, enhanced sensitivity

## Abstract

Capacitive sensors are widely adopted in compact robotic systems due to their simple structure, ease of fabrication, and scalability for miniaturized designs. However, sensor miniaturization inevitably leads to reduced sensitivity and increased sensitivity imbalance, particularly in torque measurements, due to limited electrode area and spatial constraints. To address these limitations, this paper presents a compact six-axis force/torque (F/T) sensor based on a redundant capacitive sensing architecture. The proposed sensing architecture employs a symmetric arrangement of multiple capacitive electrodes, providing redundant capacitance measurements that enhance sensitivity while reducing coupling errors under multi-axis loading conditions. By exploiting redundant capacitive responses rather than relying on complex mechanical separation, the proposed design effectively improves measurement robustness. Based on this architecture, a compact six-axis F/T sensor with a diameter of 20 mm and a height of 12 mm is developed. Experimental validation demonstrates that the proposed sensor achieves linearity (>98.2%) with reduced cross-axis interference, confirming improved sensitivity and reliable multi-axis F/T measurement. This work provides a practical and scalable solution for integrating high-performance six-axis F/T sensing into space-constrained robotic systems.

## 1. Introduction

The demand for six-axis force/torque (F/T) sensors has increased significantly across various advanced robotic applications, including robotic hand fingertips, gripper jaws, minimally invasive surgical tools, and force feedback systems. These applications rely on accurate and reliable force feedback to enable delicate manipulation, safe human-robot interaction, and precise environmental sensing in confined spaces [1,2]. Parallel mechanisms like the Stewart platform arrange one-axis sensing elements to measure six-axis F/T; however, miniaturizing these architectures entails high structural complexity and geometric alignment errors that degrade performance [3]. In contrast, conventional six-axis sensors measure six-axis F/T within a single compliant structure, offering structural simplicity and ease of miniaturization. Consequently, for successful integration into these compact robotic systems, F/T sensors must be designed with a minimized form factor while maintaining exceptional multidirectional measurement accuracy [4,5,6].

Various sensing principles have been explored to meet these requirements [7]. Strain gauge-based sensors offer high accuracy but require complex manual wiring and are sensitive to electromagnetic interference (EMI), often resulting in significant cross-axis coupling [8]. Optical sensors, such as those using Fiber Bragg Gratings (FBG), are immune to EMI; however, integrating light sources and detectors into a small space typically requires complex multilayer structures to achieve partial mechanical decoupling [9,10]. In contrast, capacitive F/T sensors offer notable advantages for miniature designs, including high sensitivity, simple structure, low power consumption, and cost-effective fabrication [11]. Despite these advantages, miniaturizing sensors has inherent challenges. A primary limitation is the reduced electrode area, which leads to lower sensitivity. More critically, strict spatial constraints make it challenging to arrange independent electrodes that physically separate normal and shear sensing directions. This spatial limitation often leads to sensitivity imbalances and severe cross-axis coupling, in which a load applied in one direction induces unintended responses in others, thereby degrading measurement reliability.

To minimize coupling errors, previous studies have primarily focused on structural decoupling methods. These approaches employ complex flexure mechanisms or multi-frame structures to mechanically isolate deformation modes for each axis [12]. Similarly, an analytical geometric synthesis method was developed to realize a diagonal stiffness matrix, enabling complete stiffness decoupling [13,14]. Structurally separating deformation paths in multi-body designs enables more independent axis responses [15]. Condition-number analysis has been widely adopted to optimize the geometry of compliant structures for stiffness isotropy. This approach facilitates the equalization of deformation responses, thereby significantly reducing cross-axis coupling.

Beyond structural design considerations, the sensing performance of capacitive sensors is also influenced by the electrode configuration. In conventional capacitive sensors, force and torque components are typically measured by arranging sensing electrodes according to the normal and shear directions of the applied loads [16]. To distinguish these directional components, electrodes are often assigned to specific force or torque components, resulting in sensor designs with six or eight electrodes [1,17]. Such configurations lead to an approximate one-by-one correspondence between each electrode and a specific loading direction [18]. The intrinsic nonlinearity of capacitive sensing leads to asymmetric responses depending on whether the electrode gap decreases or increases. This characteristic may restrict the effective use of the nonlinear sensing range. Furthermore, since electrode layouts in conventional capacitive sensors are mostly standardized, most research has focused on optimizing the compliant structure rather than the sensing architecture itself to improve performance [19,20]. While recent studies have used symmetric electrode arrays to improve sensing properties, the configuration proposed in a previous study is challenging to design compact sensors [21]. Consequently, there is still potential for further research into capacitive sensing architectures that enhance sensitivity while keeping a compact sensor design.

This paper presents a compact six-axis F/T sensor based on a redundant capacitive sensing architecture to enhance its sensitivity. This sensing architecture enables redundant electrodes to simultaneously respond to changes in capacitance induced by force and torque loads, thereby improving sensitivity through electrode interactions. In addition, the electrode’s interactive reaction is a very efficient measurement method, as all electrodes respond to the force-torque load. Unlike conventional capacitive sensor design, compact sensor design is also possible because all electrodes are integrated into the compliance structure of the proposed sensing architecture. As a result, the proposed sensor achieves compact, high-sensitivity six-axis F/T measurements, suitable for integration into space-constrained robot applications, such as gripper-jaw integration and robotic hand fingertips. This paper is organized as follows. Section 2 explains the basic capacitive sensing principle and presents the redundant capacitive sensing architecture, including its structural configuration. Section 3 presents the assembly and fabrication of the proposed compact six-axis F/T sensor. Section 4 describes an experimental validation covering the calibration process and performance evaluation. Finally, Section 5 concludes the paper and discusses the proposed sensor.

## 2. Design of the Redundant Capacitive Sensing Architecture

### 2.1. Sensing Principle

**Figure 1:**
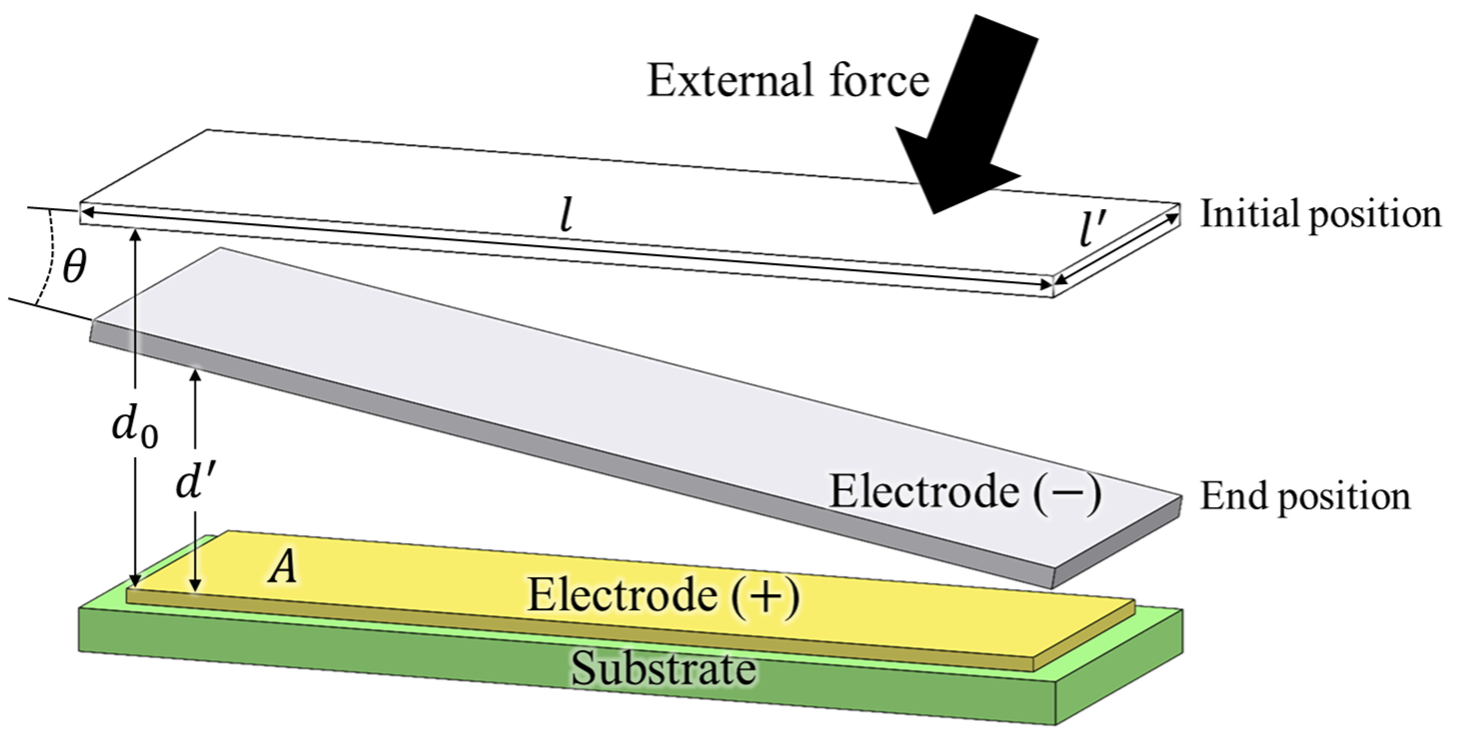
Capacitance sensing principle based on parallel plate configuration with tilting.

As illustrated in Figure 1, the fundamental sensing principle of a capacitive sensor relies on the electric field formed between two electrodes arranged in a parallel-plate configuration. In this arrangement, the capacitance is determined primarily by the overlapping electrode area and the distance between the plates. When an external force is applied to the grounded plate, it induces a relative displacement, which alters the gap distance between the electrodes, resulting in a measurable change in capacitance. This fundamental relationship is governed by the parallel-plate capacitor equation, expressed as:

(1) $$\begin{array}{c} \begin{array}{c} C=\varepsilon_{0}\varepsilon_{r}\frac{A}{d} \end{array} \end{array}$$

where Equation (1) *C* is the capacitance between the two electrodes, *A* is the overlapping electrode area, and *d* is the distance between them. $\varepsilon_{0}$ and $\varepsilon_{r}$ denote the permittivity of free space and the relative dielectric constant of air, respectively. The capacitance of the initial position can be written as

(2) $$\begin{array}{c} \begin{array}{c} C_{0}=\varepsilon_{0}\varepsilon_{r}\frac{ll^{\prime }}{d_{0}} \end{array} \end{array}$$

where Equation (2) $C_{0}$ is the capacitance in the initial position, *l* and $l^{\prime }$ denote the length and width of the grounded electrode plate, respectively, and $d_{0}$ is the initial gap between the parallel plates. In practical implementations, the sensing electrodes inside the sensor not only measure parallel displacement but also slight tilting when an external force is applied. The capacitance at the end position, considering distance and angle, can be expressed as

(3) $$\begin{array}{c} \begin{array}{c} d\left(x\right)=d^{\prime }+x\tan\theta \;,\;\left(0\leq x\leq l\right) \end{array} \end{array}$$

(4) $$\begin{array}{c} \begin{array}{c} C^{\prime }=\varepsilon_{0}\varepsilon_{r}l^{\prime }\int_{0}^{l}\frac{dx}{d^{\prime }+x\tan\theta }=\frac{\varepsilon_{0}\varepsilon_{r}l^{\prime }}{\tan\theta }ln\left(\frac{d^{\prime }+l\tan\theta }{d^{\prime }}\right) \end{array} \end{array}$$

where Equation (3) $d\left(x\right)$ denotes the local gap between the grounded plate and the sensing electrode at position *x*, $d^{\prime }$ is the separation distance, and θ represents the tilt angle of the grounded plate. The variable *x* is defined along the length direction of the grounded plate, satisfying $\left(0\leq x\leq l\right)$. As shown in Equation (4), the term $C^{\prime }$ represents the capacitance of the tilted parallel-plate configuration obtained by integrating the position-dependent gap over the effective electrode area [22]. Combining Equations (2) and (4), the capacitance variation ΔC is formulated as Equation (5). Accordingly, the capacitance variation ΔC is defined as the difference between the capacitance at the deformed end position and that at the initial configuration and can be expressed as

(5) $$\begin{array}{c} \begin{array}{c} \Delta C=C^{\prime }-C_{0}=\varepsilon_{0}\varepsilon_{r}l^{\prime }\left[\frac{1}{\tan\theta }\ln\left(\frac{d^{\prime }+l\tan\theta }{d^{\prime }}\right)-\frac{l}{d_{0}}\right],\;\left(\theta \neq 0\right) \end{array} \end{array}$$

Equation (5) clearly shows that the capacitance sensitivity mainly depends on geometric design parameters. Equation (5) describes the capacitance changes when the grounded plate undergoes tilting (θ≠0). In the specific case where the tilt angle is zero (θ=0), the displacement simplifies to a parallel translation. Consequently, the capacitance change is governed by the standard parallel-plate formula, which can be expressed as

(6) $$\begin{array}{c} \begin{array}{c} \Delta C{|}_{\theta =0}=\varepsilon_{0}\varepsilon_{r}l^{\prime }\left(\frac{l}{d^{\prime }}-\frac{l}{d_{0}}\right) \end{array} \end{array}$$

A change in the distance of the electrode plate from $d_{0}$ to $d^{\prime }$ results in a change in capacitance, as described by Equation (6). In particular, the initial distance $d_{0}$ is a key factor in determining sensitivity, as a smaller $d_{0}$ results in a larger capacitance change even for the same deformation. In addition, the deformed distance $d^{\prime }$, electrode length *l*, and width $l^{\prime }$ directly influence the magnitude of ΔC through the scaling terms. Therefore, $d_{0}$, $d^{\prime }$, *l*, and $l^{\prime }$ are the key design parameters for the sensitivity of the capacitive sensor. In practical sensor design, maintaining tight tolerances on the initial capacitive gap $d_{0}$ is crucial, as even slight variations can lead to significant deviations in sensitivity.

### 2.2. Compliant Structure of Proposed Sensing Architecture

**Figure 2:**
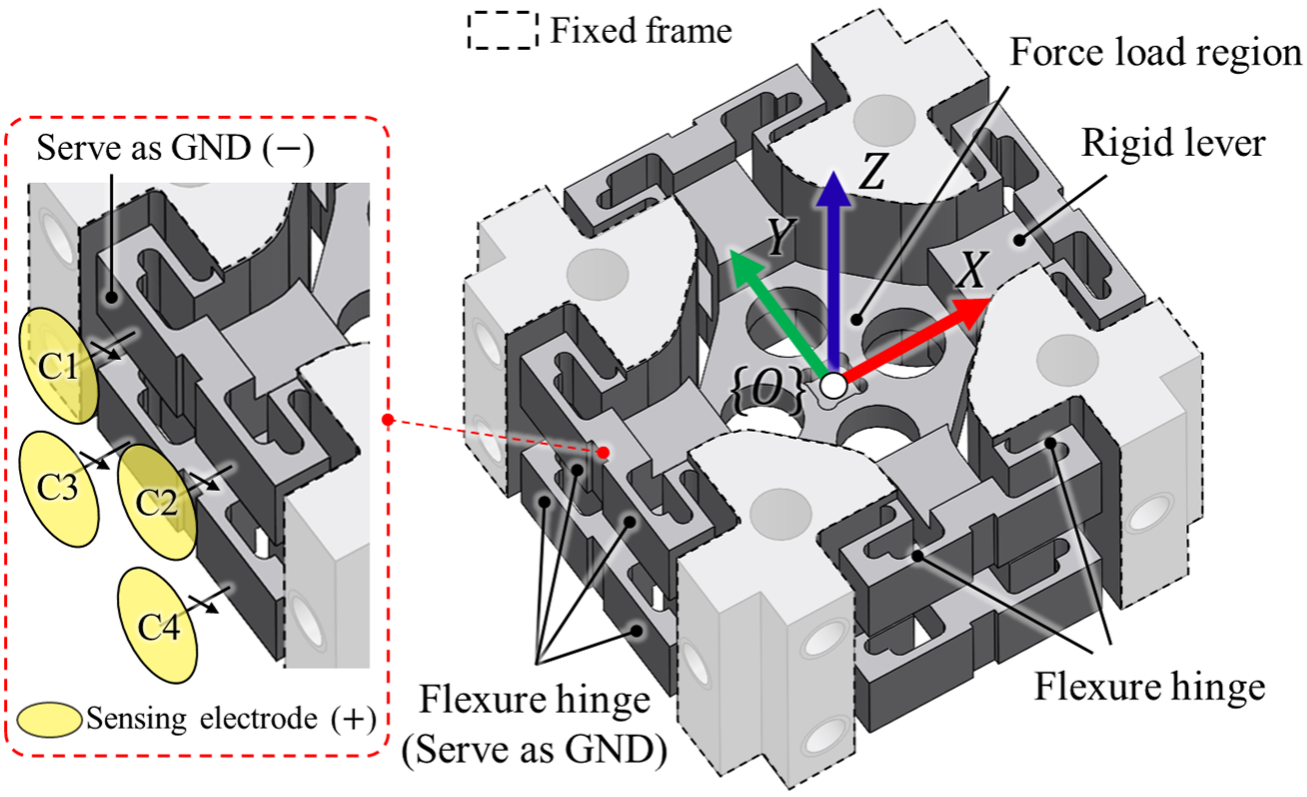
Configuration of the proposed compliant structure.

This section describes the compliant structure of the proposed redundant capacitive sensing architecture. The configuration of this structure is illustrated in Figure 2. Fixed frames are positioned at the four corners of the structure. The coordinate frame {O} is defined at the geometric center of the structure, which serves as the force loading region. When an external load is applied to this region, the force is transmitted to the flexure hinges via the rigid levers. In this configuration, the displacement occurs on the flat surface of the flexure hinge, which simultaneously serves as the GND for the capacitive sensors. As shown in the enlarged view of Figure 2, sensing electrodes are patterned to detect these displacements in capacitance. As described in Figure 1, the basic capacitance sensing principle can be composed of GND (−) and sensing electrode (+) as specified in the enlarged view on the left side of Figure 2. Notably, because the flexure hinge itself serves as GND, the proposed sensor architecture can be designed with a compact structure.

**Figure 3:**
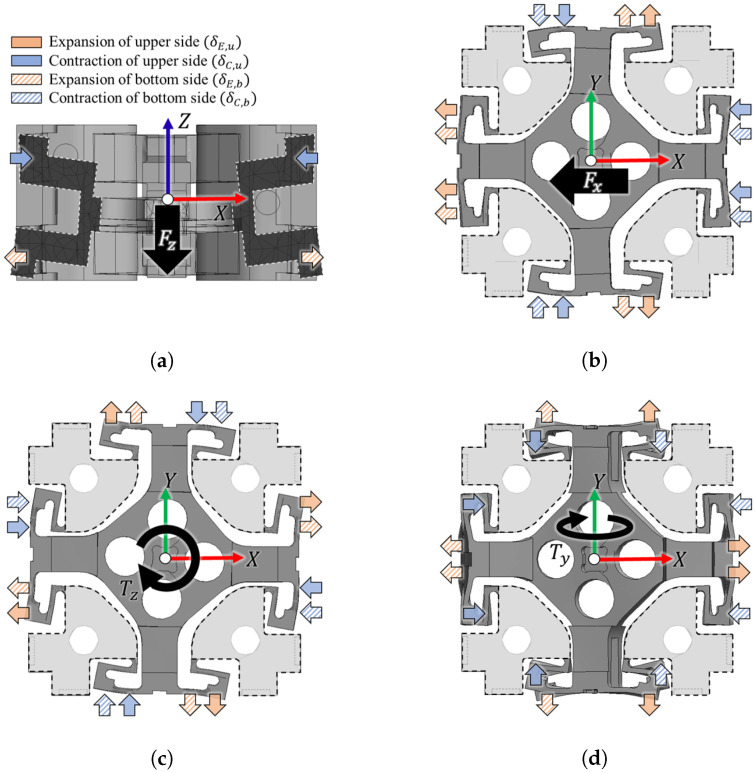
Mechanical deformation behavior of the compliant structure under various external loads: (**a**) $F_{z}$ load, (**b**) $F_{x}$ load, (**c**) $T_{z}$ load, and (**d**) $T_{y}$ load.

As illustrated in Figure 3, the compliant structure responds to external six-axis forces and torques through the expansion and contraction of the upper and bottom sides of the ground (GND) plates. Rather than moving independently, these GND plates exhibit coupled interactions, resulting in distinct deformation patterns. The arrows in the figure correspond to the legend provided in Figure 3a, which denotes the expansion ($\delta_{E}$) and contraction ($\delta_{C}$) of the upper (*u*) and bottom (*b*) sides. The structural behavior varies depending on the type of applied load. Under a $F_{z}$ load (Figure 3a), the structure deforms such that the upper side contracts while the bottom side expands. In the case of a $F_{x}$ load (Figure 3b), the plates along the *X*-axis expand and contract in alignment with the force direction, whereas the plates along the *Y*-axis exhibit a tilting motion in the orthogonal axis. This tilting behavior, induced by the structural interaction, is a critical factor in enhancing the sensitivity of the proposed redundant capacitive sensing architecture. Regarding $T_{z}$ load (Figure 3c), the structure demonstrates highly symmetric movements. Under a $T_{y}$ load (Figure 3d), the behavior is more distinctive; while the plates along the *X*-axis move symmetrically, the plates along the *Y*-axis undergo axial torsion. This torsional movement results in a unique diagonal deformation pattern where expansion and contraction occur in diagonal pairs. Similar to the tilting effect observed under lateral loads, this coupled diagonal motion plays a significant role in improving the overall sensitivity of the sensor.

### 2.3. Mechanical Analysis

**Figure 4:**
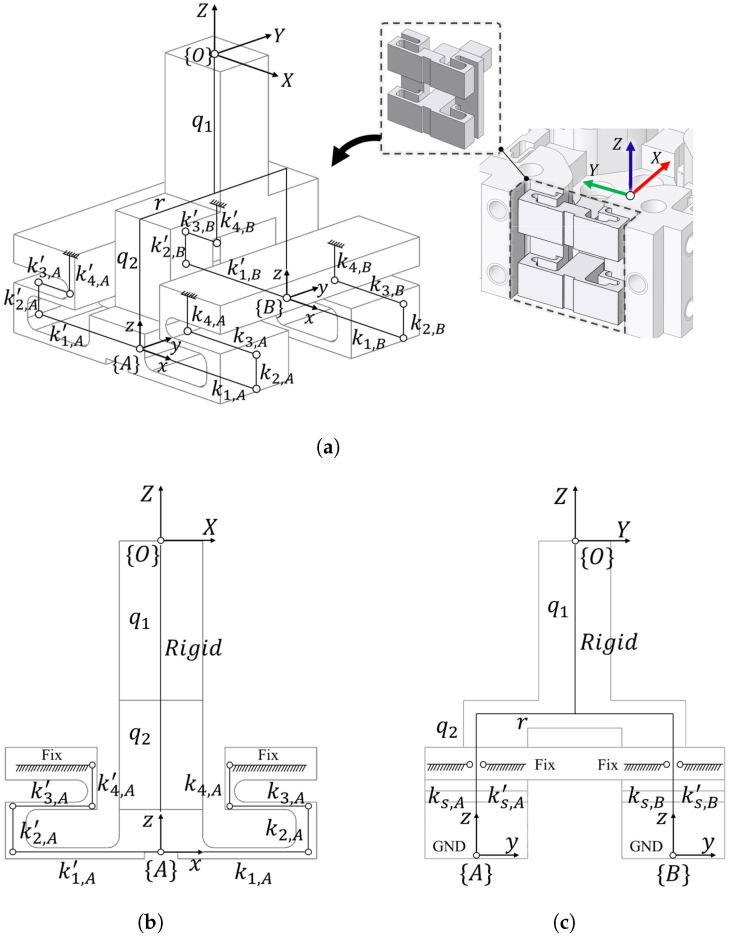
Mechanical modeling of the compliant structure based on its symmetric configuration. (**a**) Schematic of the representative substructure isolated for stiffness analysis. (**b**) Simplified mechanical model of frame {A} on the *X*-*Z* plane. (**c**) Simplified mechanical model of the combined local frame {A} and {B} on the *Y*-*Z* plane.

Mechanical analysis of the displacement induced by six-axis forces and torques is a prerequisite for designing the proposed redundant capacitive sensing architecture. Given the symmetric configuration of the compliant structure, the stiffness analysis can be simplified by isolating a single representative substructure. As illustrated in Figure 4a, this substructure consists of symmetric flexure hinges defined within local frames {A} and {B}, which are characterized by stiffness parameters $\left(k_{1},k_{2},k_{3},k_{4}\right)$ and $\left(k_{1}^{\prime },k_{2}^{\prime },k_{3}^{\prime },k_{4}^{\prime }\right)$, respectively. A global reference frame {O} is defined at the top of the rigid link, serving as the point where external forces are applied. The applied load is then transmitted through the rigid link, whose geometry is defined by the parameters $q_{1}$, $q_{2}$, and *r*. Owing to the symmetric arrangement of the flexure hinges, the resulting displacement of the grounded plate is selectively constrained to specific translational and rotational movement. For stiffness analysis, the three-dimensional structure is reduced to the equivalent two-dimensional models, as shown in Figure 4b,c. Note that due to the structural symmetry, the mechanical behaviors of local frames {A} and {B} are identical in projection. Thus, they can be represented as a combined equivalent model on the *Y*-*Z* plane without loss of generality. In this analysis, the rigid link is assumed to be non-deformable, and all displacements are concentrated in the flexure hinges [23,24]. Under the small-deformation assumption, the proposed compliant structure can be mechanically modeled as a linear elastic system. Accordingly, the external load and the resulting displacement are related by the following linear relationship:

(7) $$\begin{array}{c} \begin{array}{cc} f & =Kd \end{array} \end{array}$$

(8) $$\begin{array}{cc} f & ={\left[\begin{array}{cccc} F_{x} & F_{y} & F_{z} & T_{z} \end{array}\right]}^{T},\;d={\left[\begin{array}{cccccc} \Delta x_{A} & \Delta z_{A} & \theta_{y,A} & \Delta x_{B} & \Delta z_{B} & \theta_{y,B} \end{array}\right]}^{T} \end{array}$$

where K is the stiffness matrix of the compliant structure, $F_{x}$, $F_{y}$, $F_{z}$, $T_{z}$ denote the applied wrench f at the reference frame, and $\Delta x_{A}$, $\Delta z_{A}$, $\Delta x_{B}$, $\Delta z_{B}$ represent the resulting displacement vector d at frame {A} and {B} respectively, and $\theta_{y,A}$, $\theta_{y,B}$ are the rotational displacements. In the following Equation (8), the torque components $T_{x}$ and $T_{y}$ are not considered. This is because the displacement patterns derived from simplified machine models are similar to those generated by $F_{x}$ and $F_{y}$, as well as by $T_{x}$ and $T_{y}$. When the model shown in Figure 4 is isotropically arranged, the behavior of the external six-axis F/T is decomposed into the force components $F_{x},F_{y},F_{z}$ and the axial torque $T_{z}$ for each simplified mechanical model. Therefore, in the simplified model, the displacement patterns in response to the four axes of $F_{x},F_{y},F_{z}$ and $T_{z}$ should be identified analytically. In particular, a $F_{y}$ applied at {O} generates a bending moment about the *x*-axis with an effective lever arm given by

(9) $$\begin{array}{c} \begin{array}{c} q=q_{1}+q_{2} \end{array} \end{array}$$

which leads to differential vertical displacements at frame {A} and {B}. Similarly, an applied torque $T_{z}$ is balanced by opposing reactions at the flexure hinge, separated by a distance *r*, resulting in differential displacement along the *z*-axis. These geometric couplings are inherently reflected in the stiffness matrix K through the corresponding lever-arm terms, thereby establishing a direct relationship between the applied loads and the measured displacements.

(10) $$\begin{array}{c} \begin{array}{c} k_{s,i}={\left(\sum_{j=1}^{4}\frac{1}{k_{j,i}}\right)}^{-1},\;k_{s,i}^{\prime }={\left(\sum_{j=1}^{4}\frac{1}{k_{j,i}^{\prime }}\right)}^{-1},\;i\in A,B \end{array} \end{array}$$

According to Equation (10), the equivalent stiffness $k_{s,i}$ and $k_{s,i}^{\prime }$ are obtained by combining the stiffnesses of the four flexure hinges arranged in the structure. Due to the series arrangement of the hinges along the deformation path, the equivalent stiffness can be expressed as the reciprocal sum of the individual hinge stiffnesses.

(11) $$\begin{array}{c} \begin{array}{c} K_{s,A}=k_{s,A}+k_{s,A}^{\prime }\;,\;K_{s,B}=k_{s,B}+k_{s,B}^{\prime } \end{array} \end{array}$$

As illustrated in Figure 4c, the stiffnesses of frames {A} and {B} can be expressed by Equation (11). Each frame is represented as a linearly elastic, compliant element that generates restoring forces proportional to the applied displacement.

(12) $$\begin{array}{c} F_{x,i}=K_{s,i}\Delta x_{i}^{\mathrm{eff}},\;F_{z,i}=K_{s,i}\Delta z_{i}^{\mathrm{eff}},\;i\in A,B \end{array}$$

where $K_{s,i}$ is the equivalent stiffness of frame *i*, $\Delta x_{i}^{\mathrm{eff}}$ and $\Delta z_{i}^{\mathrm{eff}}$ are the translational displacements of frame *i* along the *x* and *z*-axes, respectively. The effective displacement along the *x*-direction accounts for the rotational contribution and is defined as

(13) $$\Delta x_{i}^{\mathrm{eff}}=\Delta x_{i}+q\theta_{y,i}$$

with $\theta_{y,i}$ denoting the rotation about the *y*-axis and *q* as the corresponding lever arm. Note that the displacement along the *z*-direction is not affected by the rotational motion under the proposed kinematic configuration. Based on the static equilibrium of frames {A} and {B}, the external wrench is linearly related to the generalized displacement vector by Equation (7). The resultant force along the *x*-direction is given by

(14) $$F_{x}=K_{s,A}\left(\Delta x_{A}+q\theta_{y,A}\right)+K_{s,B}\left(\Delta x_{B}+q\theta_{y,B}\right)$$

while the force along the *z*-direction is expressed as

(15) $$F_{z}=K_{s,A}\Delta z_{A}+K_{s,B}\Delta z_{B}$$

The force component $F_{y}$ is derived from the torque equilibrium about the lever arm *q*, balanced by the couple generated by the vertical reaction forces separated by a distance *r*.

(16) $$F_{y}=\frac{r}{q}\left(K_{s,B}\Delta z_{B}-K_{s,A}\Delta z_{A}\right)$$

Finally, the torque about the *z*-axis is obtained as

(17) $$T_{z}=r\left[K_{s,B}\left(\Delta x_{B}+q\theta_{y,B}\right)-K_{s,A}\left(\Delta x_{A}+q\theta_{y,A}\right)\right]$$

These relations collectively define the stiffness matrix K of the proposed compliant structure. By rearranging the equilibrium equations into the form f=Kd, the explicit expression of the stiffness matrix is obtained as

(18) $$\left[\begin{array}{c} F_{x}\\ F_{y}\\ F_{z}\\ T_{z} \end{array}\right]=\left[\begin{array}{cccccc} K_{s,A} & 0 & qK_{s,A} & K_{s,B} & 0 & qK_{s,B}\\ 0 & -\frac{r}{q}K_{s,A} & 0 & 0 & \frac{r}{q}K_{s,B} & 0\\ 0 & K_{s,A} & 0 & 0 & K_{s,B} & 0\\ -rK_{s,A} & 0 & -rqK_{s,A} & rK_{s,B} & 0 & rqK_{s,B} \end{array}\right]\left[\begin{array}{c} \Delta x_{A}\\ \Delta z_{A}\\ \theta_{y,A}\\ \Delta x_{B}\\ \Delta z_{B}\\ \theta_{y,B} \end{array}\right]$$

The stiffness matrix K explicitly captures the linear mapping from the generalized deformations of the two frames to the resulting external forces and torques. According to Equation (18), the compliance matrix can be expressed as

(19) $$d=Cf,\;C=K^{T}{\left(KK^{T}\right)}^{-1}$$

(20) $$\left[\begin{array}{c} \Delta x_{A}\\ \Delta z_{A}\\ \theta_{y,A}\\ \Delta x_{B}\\ \Delta z_{B}\\ \theta_{y,B} \end{array}\right]=\left[\begin{array}{cccccc} \frac{1}{2K_{s,A}} & 0 & 0 & 0 & 0 & 0\\ 0 & \frac{1}{2K_{s,A}} & 0 & 0 & 0 & 0\\ 0 & 0 & \frac{1}{2K_{s,A}} & 0 & 0 & 0\\ 0 & 0 & 0 & \frac{1}{2K_{s,B}} & 0 & 0\\ 0 & 0 & 0 & 0 & \frac{1}{2K_{s,B}} & 0\\ 0 & 0 & 0 & 0 & 0 & \frac{1}{2K_{s,B}} \end{array}\right]\left[\begin{array}{cccc} \frac{1}{1+q^{2}} & 0 & 0 & -\frac{1}{r(1+q^{2})}\\ 0 & -\frac{q}{r} & 1 & 0\\ \frac{q}{1+q^{2}} & 0 & 0 & -\frac{q}{r(1+q^{2})}\\ \frac{1}{1+q^{2}} & 0 & 0 & \frac{1}{r(1+q^{2})}\\ 0 & \frac{q}{r} & 1 & 0\\ \frac{q}{1+q^{2}} & 0 & 0 & \frac{q}{r(1+q^{2})} \end{array}\right]\left[\begin{array}{c} F_{x}\\ F_{y}\\ F_{z}\\ T_{z} \end{array}\right]$$

Equations (19) and (20) establish the compliance matrix, which characterizes the elastic relationship between the applied wrench and the induced structural displacement.

### 2.4. Redundant Capacitive Sensing Architecture

**Figure 5:**
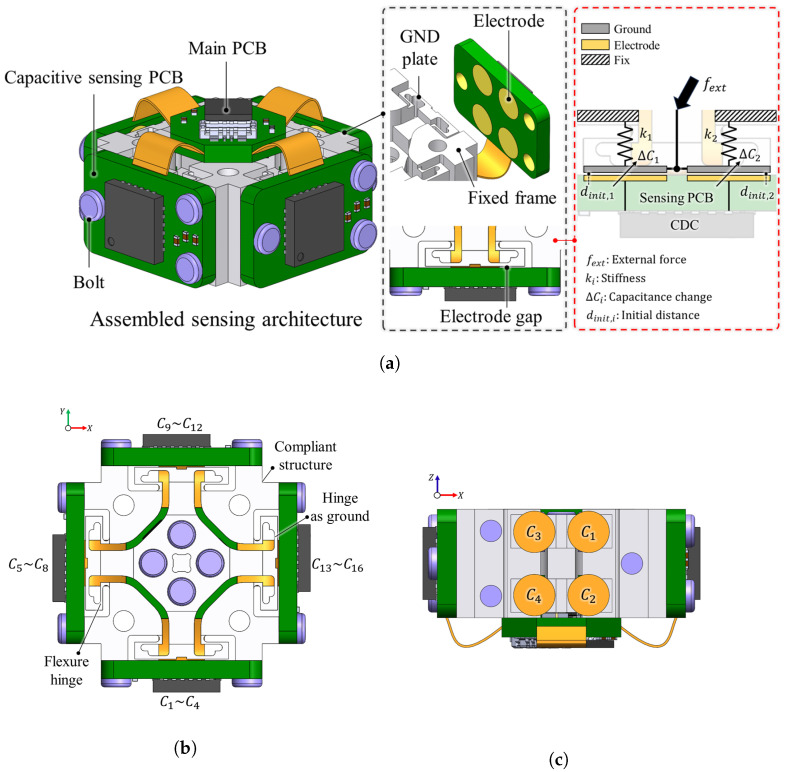
Proposed redundant capacitive sensing architecture. (**a**) Fully assembled sensing architecture and configuration. (**b**) Top view illustrating the layout of the 16 electrodes $(C_{1}$–$C_{16})$. (**c**) Side view displaying the placement of the 4 electrodes $(C_{1}$–$C_{4})$.

Based on the mechanical deformation characteristics analyzed in the previous section, we propose a Redundant Capacitive Sensing Architecture (RCSA). This architecture is strategically designed to measure displacements in the compliant structure directly with capacitive changes. This redundancy plays a key role in reducing sensitivity loss and manufacturing inconsistencies, thereby significantly improving the sensitivity and linearity of the miniaturized sensor [25,26]. The RCSA features a spatially orthogonal electrode array aligned with the Cartesian *X*-*Y* axes as shown in Figure 5. This configuration not only simplifies the coordinate definitions and transformation matrices in the sensor model but also facilitates ease of fabrication [27]. A key feature of this design is to leverage the structure’s tolerance to maintain a consistent initial capacitance distance. Unlike traditional sensor designs that require spatially constrained elements to separate the electrode arrays along the direction, the proposed architecture leverages the precision of symmetric components to achieve the target spacing through simple bolting assembly. As illustrated in Figure 5a, the fully assembled RCSA comprises the compliant structure, a main PCB, and capacitive sensing PCBs. To ensure reliable signal transmission while maintaining structural compactness, the main and sensing PCBs are designed using rigid-flex PCB, allowing them to be flexibly interconnected without mechanical interference. As shown in the expanded view of Figure 5a (right side), the sensing PCBs are mounted to the structure’s fixed frame. An advantage of this assembly strategy is the passive formation of the initial capacitive gap. A precise 100 μm machining tolerance is designed between the frame and the GND plate. Consequently, the initial gap ($d_{i}nit$) between the electrode and the GND plate is naturally established through simple bolting, eliminating the need for complex alignment. In traditional designs, keeping a consistent $d_{init}$ usually requires extra spacers, making assembly more complicated. When an external force ($f_{ext}$) is applied, the compliant structure represented by stiffness elements $k_{1}$ and $k_{2}$ undergoes displacement. This displacement directly changes the electrode gaps $d_{init}$, causing changes in capacitance (ΔC). The 16 electrodes ($C_{1}$–$C_{16}$) detect these displacements as unique combinations of synchronous or differential responses, enabling the sensing of six-axis F/T. Figure 5b presents the top view of the architecture, demonstrating the isotropic arrangement, which contributes to the structural stability of the overall assembly. Because of the entire conductive compliant structure to act as the common GND electrode. Specifically, some hinges of the flexure hinge configuration can act as the GND of the sensing configuration. Consequently, there is no need to fabricate or arrange a separate GND component, thereby significantly contributing to the sensor’s compact configuration. Furthermore, Figure 5c provides the side view, detailing the specific spatial arrangement and numbering of the electrodes ($C_{1}$–$C_{4}$) relative to the upper and lower surfaces of the GND plate. Consequently, the proposed RCSA significantly enhances the sensor’s measurement sensitivity. Although the capacitance changes caused by structural displacement exhibit coupled interactions, the specific combination of these responses remains independent in each of the six axes of force and torque. This independence ensures that the applied loads can be distinctly resolved, thereby minimizing cross-axis coupling errors.

The proposed redundant capacitive sensing architecture offers distinct advantages over conventional designs. First, the redundant arrangement of sensing elements enables signal stabilization and enhanced sensitivity. Sensitivity increases because the sensing elements respond not only to the direction of the applied load but also to other axial directions. Second, the architecture overcomes structural limitations to achieve robust decoupling. Even without an optimized isotropic design or mechanically decoupled structure, the redundant array generates unique sensing motions and distinguishable signal patterns for all six axes, ensuring robustness against coupling errors. Third, by using the flexure hinge itself as a GND, a compact and robust sensor can be implemented without additional parts.

### 2.5. FEA Simulation

**Figure 6:**
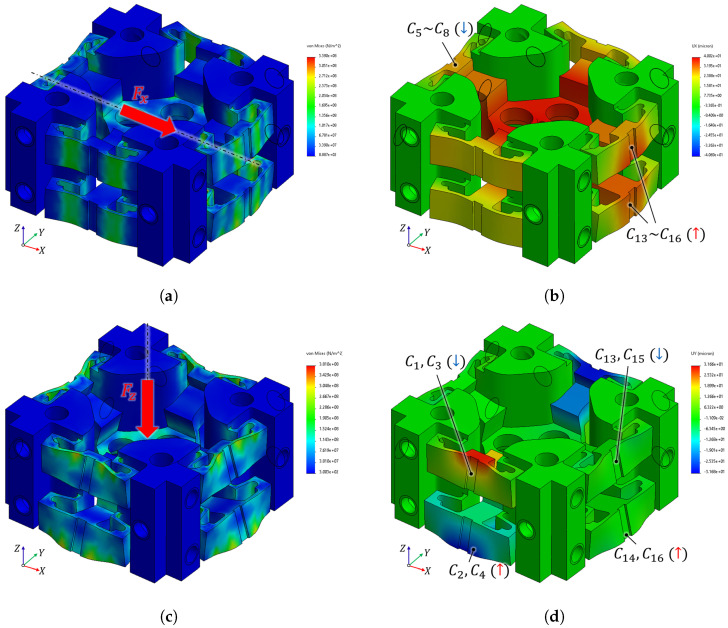
FEA simulation results. (**a**) Von Mises stress distribution under $F_{x}$. (**b**) *X*-directional displacement ($U_{x}$) under $F_{x}$. (**c**) Von Mises stress distribution under $F_{z}$. (**d**) *Y*-directional displacement ($U_{y}$) induced by $F_{z}$.

Finite Element Analysis (FEA) is performed using SolidWorks Simulation 2021 (Dassault Systèmes, USA) to validate the compliant structure’s stiffness and displacement, and to assess its structural safety. A linear static analysis is performed assuming small deformations within the elastic region. The structure is analyzed using a standard solid mesh of second-order tetrahedral elements, with a mesh size of approximately 0.40 mm and a mesh tolerance of 0.02 mm. The material properties of aluminum alloy 7075-T6 (Hyeokjun Industry, Gimhae-si, Republic of Korea; Young’s modulus: 71.7 GPa, Poisson’s ratio: 0.33, Yield strength: 503 MPa) were assigned, selected for its high strength-to-weight ratio and low hysteresis. A primary objective of this simulation is to verify how the mechanical deformation translates into distinct capacitive changes within the proposed RCSA. Figure 6a presents the Von Mises stress distribution under a maximum rated $F_{x}$ load of 100 N. The simulation indicates a peak stress of 340 MPa, which is strictly localized at the flexure hinges. Given the yield strength of 503 MPa, this corresponds to a safety factor of approximately 1.48, confirming that the structure maintains sufficient durability within the linear elastic region. Figure 6b illustrates the resulting displacement under the $F_{x}$ load. The structure exhibits a lateral displacement ($U_{x}$) of approximately 40μm, aligned with the force vector. As the structure deforms, the symmetrically arranged capacitive electrodes exhibit opposite trends: the electrode gaps in the loading direction ($C_{13}$–$C_{16}$) decrease, while those in the opposing direction ($C_{5}$–$C_{8}$) simultaneously increase. Furthermore, the electrodes aligned along the Y-axis also exhibit a simultaneous response. This coupled behavior is not parasitic but designed to actively contribute to the total capacitance change, thereby enhancing the measurement sensitivity for $F_{x}$.

Regarding the normal force ($F_{z}$) case, the structural safety is evaluated in Figure 6c. The simulation indicates a peak stress of 380 MPa under the maximum rated load. Although this stress level is slightly higher than that observed in the shear load case, it yields a safety factor of approximately 1.32, confirming that the structure remains safely within the linear elastic region. Figure 6d illustrates the unique displacement characteristic of the compliant structure in response to $F_{z}$. Unlike conventional sensors, which usually respond to displacements in the same direction as the axial direction, the proposed design converts the applied vertical load ($F_{z}$) into lateral shear displacements ($U_{y}$). This force-to-displacement conversion causes a distinct differential gap change: the gaps in the upper side electrode ($C_{1},C_{3}$) decrease, while those in the bottom side electrode ($C_{2},C_{4}$) increase simultaneously. Consequently, the $F_{z}$ loading case results in a mechanical response that is fundamentally different from that under other axes, effectively ensuring the independence of the $F_{z}$ measurement.

**Figure 7:**
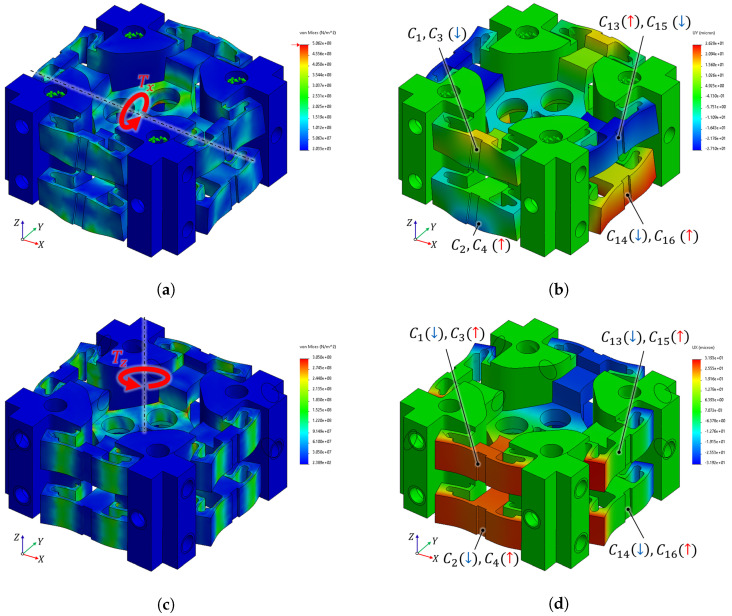
FEA simulation results. (**a**) Von Mises stress distribution under $T_{x}$. (**b**) *Y*-directional displacement ($U_{y}$) under $T_{x}$. (**c**) Von Mises stress distribution under $T_{z}$. (**d**) *X*-directional displacement ($U_{x}$) induced by $T_{z}$.

The structural safety under a torque load ($T_{x}$) is analyzed in Figure 7a. At the maximum rated torque of 0.5 Nm, the simulation indicates a relatively high stress concentration at the flexure hinges, approaching the material’s yield strength. To maximize sensitivity in the miniaturized sensor design, a lower safety factor range (1.0–1.5) is used as a deliberate engineering trade-off. Nevertheless, the peak stress remains strictly bounded within the linear elastic region, ensuring that no plastic deformation occurs even under maximum load conditions. Figure 7b illustrates the corresponding displacement behavior. The structure exhibits a *Y*-directional displacement ($U_{y}$) of approximately ±30μm. The grounded plates positioned along the *X*-axis undergo a twisting deformation, resulting in a differential capacitive response where the capacitance values of $C_{13}$ and $C_{16}$ increase, while $C_{14}$ and $C_{15}$ decrease. Simultaneously, the plates along the transverse *Y*-axis exhibit a paired movement of their upper and bottom sides. This phenomenon demonstrates that the grounded plates on both the *X* and *Y* axes operate in a complementary manner under the applied torque. Consequently, this coupled interaction actively contributes to the total capacitance change, serving as a critical factor in enhancing the measurement sensitivity for $T_{x}$.

Regarding the torque load ($T_{z}$) case, the structural safety is evaluated in Figure 7c. Under the same rated load of 0.5 Nm, the simulation indicates a peak stress of 305 MPa. This results in a safety factor of approximately 1.65, validating that the structure maintains a robust safety margin against twisting loads. Figure 7d illustrates the distinct displacement induced by $T_{z}$. The applied torque results in a displacement ($U_{x}$) of approximately ±32μm in the GND plate. This deformation results in a highly symmetric capacitive response. Specifically, for the plates along the *X*-axis, the capacitances $C_{1}$ and $C_{2}$ decrease, while those of $C_{3}$ and $C_{4}$ increase. Simultaneously, the plates positioned along the *Y*-axis demonstrate a corresponding behavior, where $C_{13}$ and $C_{14}$ decrease, and $C_{15}$ and $C_{16}$ increase. This global symmetry ensures that the resulting capacitance change is uniform, allowing the $T_{z}$ torque to be clearly differentiated from other loading conditions.

Based on the results from the FEA, the capacitive response characteristics of the RCSA under six-axis loading conditions are summarized in Table 1. This table highlights the distinctive differential patterns for each load case, confirming the sensor’s capability to decouple multi-axis forces and torques. Given the geometric symmetry, the behaviors of $F_{y}$ and $T_{y}$ mirror those of $F_{x}$ and $T_{x}$. Consequently, these results are not presented here.

## 3. Compact Six-Axis F/T Sensor

**Figure 8:**
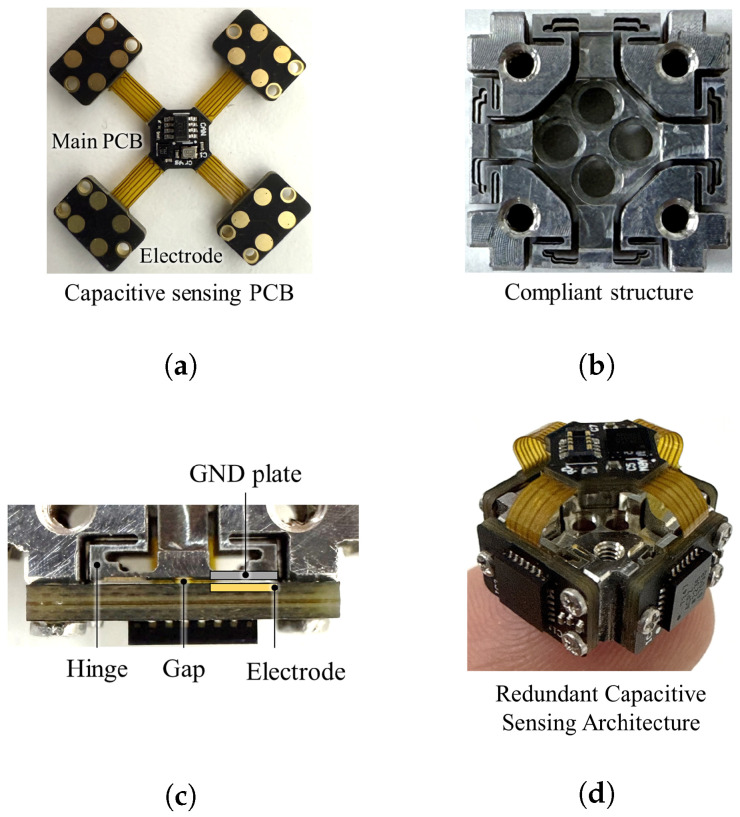
Components and assembly of the proposed RCSA. (**a**) Capacitive sensing PCB integrating four electrode arrays and the main PCB. (**b**) Compliant structure. (**c**) Close-up view of a single sensing element, illustrating the hinge, electrode, ground plate, and the initial electrode gap. (**d**) Fully assembled.

The proposed sensor architecture integrates a mechanical compliant structure with a capacitive sensing PCB, as shown in Figure 8. Figure 8a illustrates the capacitive sensing PCB, which incorporates four distributed electrode arrays fabricated using standard PCB manufacturing processes and connected to the main PCB, which plays the signal processing role. Figure 8b shows the manufactured compliant structure with dimensions of (10×10 mm) Figure 8c presents a close-up view of the assembled compliant structure and sensing PCB. Because of the precise manufacturing tolerances of the structural plate and frame, the sensor can be assembled with the ideal air gap. The fully assembled prototype of the RCSA is depicted in Figure 8d.

**Figure 9:**
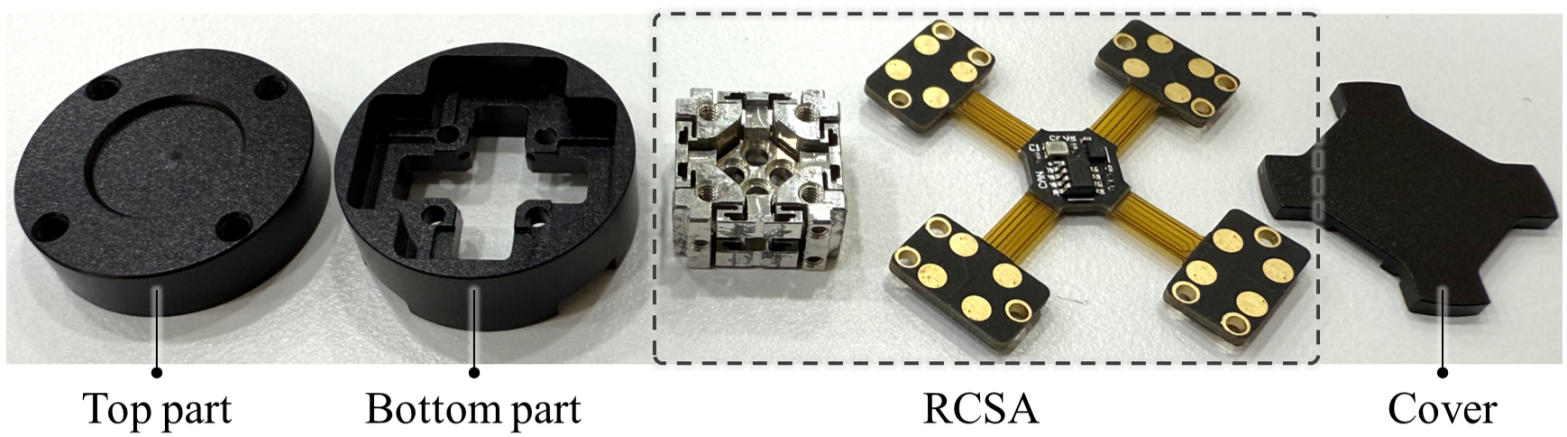
Housing components (top part, bottom part, RCSA, and cover).

After assembling the RCSA, the architecture is enclosed in a housing to ensure structural protection and support mechanical load, as shown in Figure 9. The housing components consist of a top part, a bottom part, and a rear cover, which are precisely machined from aluminum alloy (Al 6061) to ensure high structural stiffness.

**Figure 10:**
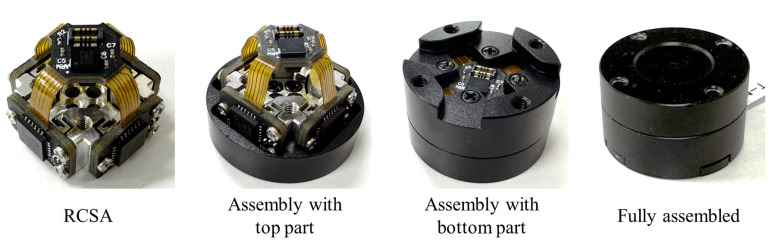
Assembly process of the proposed compact six-axis F/T sensor.

Figure 10 illustrates the final assembly process of the proposed compact six-axis F/T sensor. First, the assembled RCSA is mounted to the top part, which serves as the external force and torque. Subsequently, the bottom part is attached to the RCSA’s fixed frame. Finally, a rear cover is attached, completing the sensor fabrication. This simple and efficient fabrication and assembly process results in a highly compact sensor, measuring only 20 mm in diameter and 12 mm in height. This form factor is particularly advantageous for seamless integration into robotic end-effectors and gripper fingertips. The sensor has a measurement range of ±100 N for forces and ±0.5 Nm for torques. Furthermore, because of the integrated capacitive sensing architecture and efficient signal processing, the sensor achieves a data sampling rate of 300 Hz, as shown in Table 2.

## 4. Experimental Validation

### 4.1. Calibration

**Figure 11:**
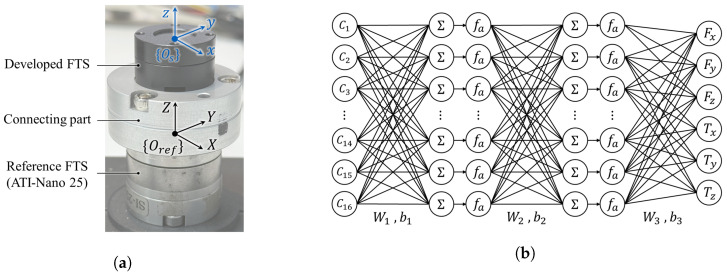
Experimental configuration for calibration and NN model. (**a**) Calibration setup with a reference sensor. (**b**) NN model for sensor calibration.

Figure 11a shows the experimental setup used for calibrating the proposed six-axis F/T sensor. The developed sensor is mounted on a commercial reference F/T sensor (ATI-Nano25, ATI Industrial Automation, Apex, NC, USA) via a rigid connecting part, allowing external forces and torques to be applied simultaneously to both sensors. This configuration enables the accurate collection of paired datasets comprising raw capacitance signals and corresponding reference F/T data under various loading conditions. Since the reference and developed F/T sensors use different coordinate frames, the measured force and torque from the reference sensor are transformed into the coordinate frame of the developed sensor before validation. The coordinates of the reference sensor $O_{ref}$ and the developed sensor $O_{s}$ are matched by referring to the coordinate system transformation formula described in [28].

In conventional calibration approaches, the least-squares method (LSM) is widely used due to its simplicity. However, LSM assumes linearity and axis-wise independence of sensor outputs, which are difficult to satisfy in a capacitive sensor. In practice, the nonlinear relationships among capacitance variation, hysteresis, and complex cross-axis coupling lead to significant calibration errors when linear mapping is applied. To address these limitations, a neural network (NN)-based calibration approach is adopted in this work [29]. As illustrated in Figure 11b, a NN is used to calibrate raw capacitance changes to six-axis force and torque outputs. The input layer of the network consists of sixteen raw capacitance change signals, $C_{1}$–$C_{16}$, obtained from the developed sensor, which are represented as $\Delta C\in R^{16}$. The network comprises two hidden layers and one output layer. The hidden layers are parameterized by weight matrices $W_{1}\in R^{16\times 16}$ and $W_{2}\in R^{16\times 16}$, with corresponding bias vectors $b_{1}\in R^{1\times 16}$ and $b_{2}\in R^{1\times 16}$. The output layer is defined by $W_{3}\in R^{16\times 6}$ and $b_{3}\in R^{1\times 6}$. A logistic sigmoid function $f_{a}$ is employed as the activation function in the hidden layers to model the nonlinear relationship. The six-axis force and torque, $F_{est}\in R^{6}$, is obtained by forward propagation of the input capacitance changes through the network using the optimized weights and biases. The parameters $\left\{W_{n},b_{n}\right\}$(n=1,2,3) are optimized during the calibration and are subsequently used for F/T output. For the calibration process, a total of 39,401 experimental data points were used. This dataset is randomly divided into training, validation, and testing sets with ratios of 70%, 15%, and 15%, respectively. The NN is trained for 2000 epochs to minimize the mean squared error.

### 4.2. Evaluation and Result

**Figure 12:**
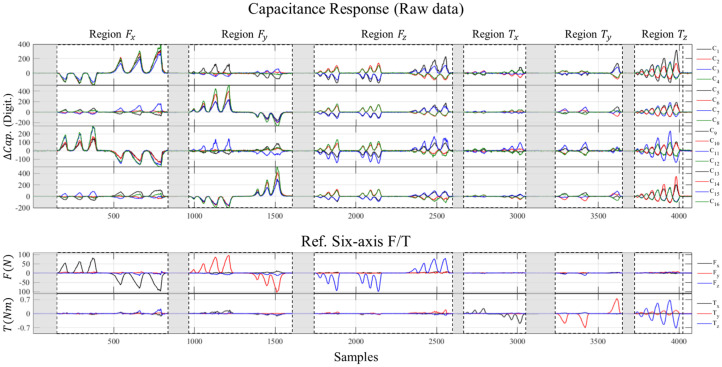
Experimental data of the capacitance response corresponding to the reference six-axis F/T loads.

To validate the feasibility of the proposed sensor, the experimental responses of the capacitive sensing electrodes corresponding to the reference six-axis loads are analyzed. Figure 12 presents the raw capacitance variations ($C_{1}$–$C_{16}$) under step-wise loading conditions for each axis. As designed in the RCSA, it is observed that specific sensing electrodes respond selectively depending on the direction of the applied force and torque. For instance, the $F_{z}$ induces a uniform change across all electrodes, whereas torques generate differential signal patterns. This result experimentally confirms that the proposed redundant sensing architecture effectively captures the decoupled deformation behavior of the compliant structure. It is also experimentally confirmed that capacitance changes due to $T_{xy}$ or $T_{z}$ loads showed a response similar to that specified in Section 2 and Table 1.

**Figure 13:**
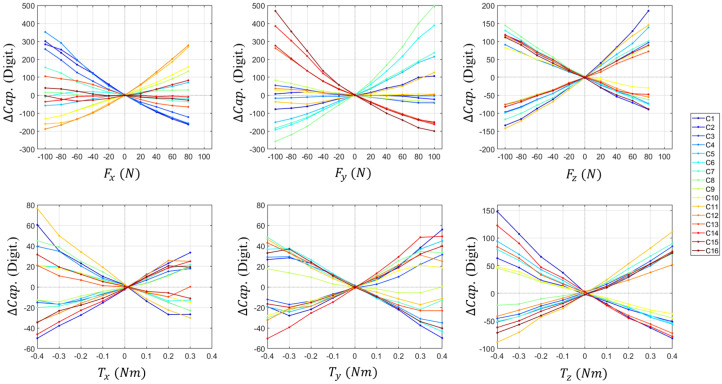
Capacitance changes of the 16-cell under a Ref. six-axis F/T load.

As shown in Figure 13, the capacitance changes of the 16 cells exhibit non-linear characteristics under the applied six-axis F/T. To address these complex non-linear responses, the raw data are processed using the parameters $W_{n}$ and $b_{n}$, derived from an NN-based calibration method, to produce linearized F/T outputs.

**Figure 14:**
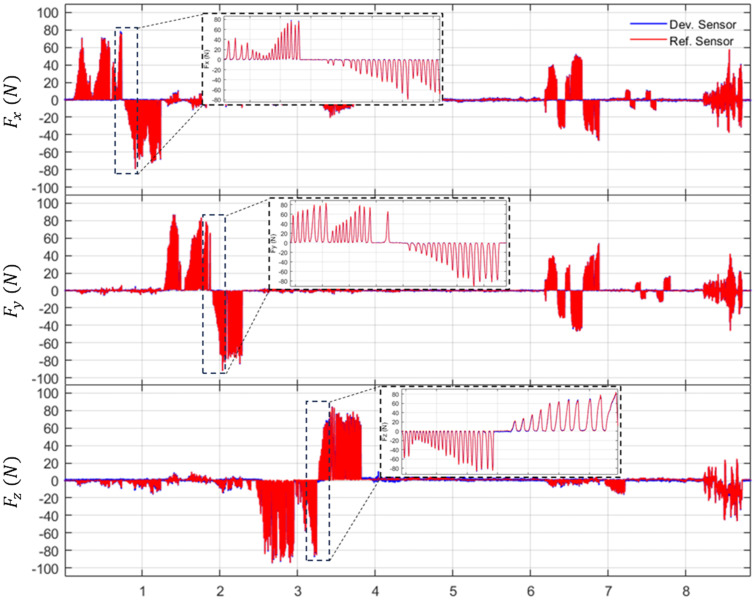
Evaluated results of forces comparison with reference sensor.

**Figure 15:**
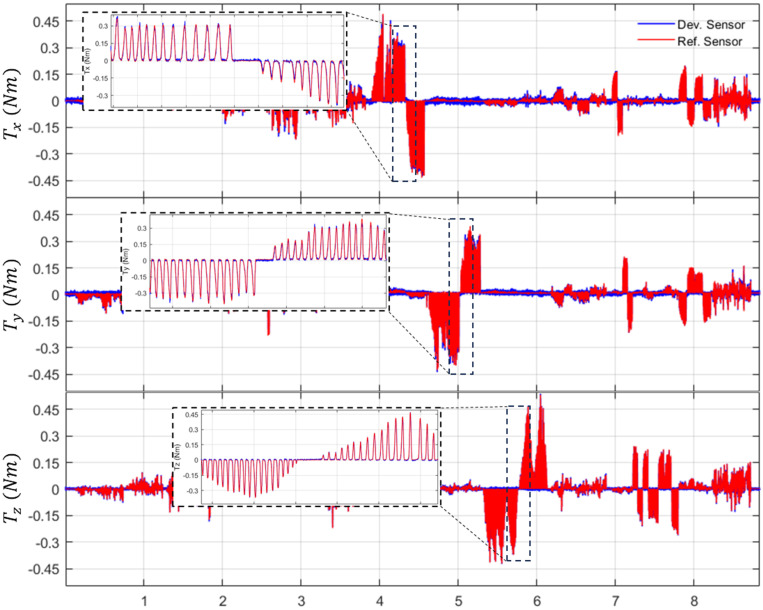
Evaluated results of torques comparison with reference sensor.

Based on these capacitive responses, the calibrated force and torque outputs of the developed sensor are compared with those of the reference sensor. Figure 14 and Figure 15 illustrate the comparison results for three-axis forces ($F_{x},F_{y},F_{z}$) and three-axis torques ($T_{x},T_{y},T_{z}$), respectively. The blue lines represent the estimated force and torque values from the developed sensor calibrated using the NN. In contrast, the red lines indicate the reference sensor. The results demonstrate that the developed sensor accurately tracks the reference signals across various loading steps, exhibiting high signal stability.

**Figure 16:**
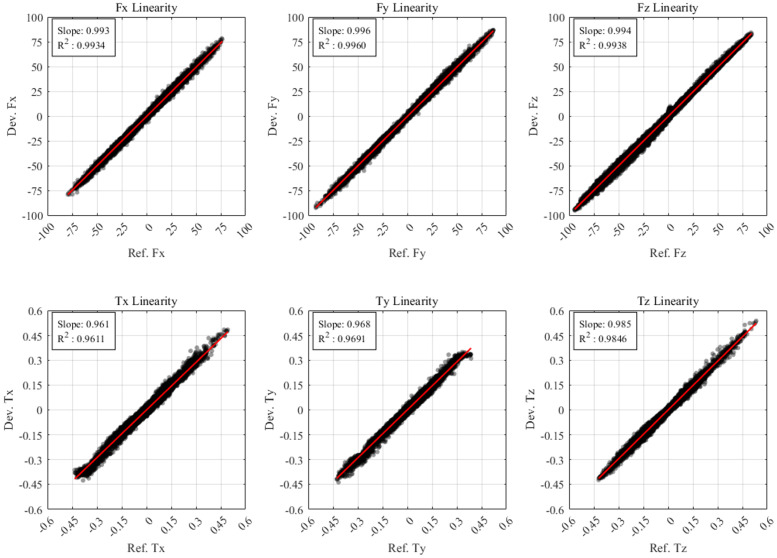
Linearity result between the reference sensor and developed sensor.

To quantitatively assess measurement reliability, the sensor’s linearity is analyzed. Figure 16 shows the scatter plots of the developed sensor output versus the reference sensor. The solid red lines represent the linear regression fit, and the coefficient of determination ($R^{2}$) is calculated for each axis. As shown in the Figure 16, all axes exhibit highly linear responses, indicating linearity and consistent sensitivity across the measurement range. Finally, the performance results of the developed six-axis F/T sensor are summarized in Table 3.

Based on the sensor evaluation method described in [30], each performance indicator in Table 3 is derived from data obtained by manually loading the proposed and reference sensors. Linearity and accuracy are calculated via linear regression of the input–output data over the full range of applied loads. The proposed sensor shows a linearity range from 98.20% ($T_{y}$) to 99.25% ($F_{y}$). The sensor presents a maximum accuracy error of 0.96% ($F_{z}$) and a minimum error of 0.04% ($F_{y}$). Crosstalk represents the maximum error occurring on the other axes when F/T is applied to a single axis, with the overall maximum error being 1.70% ($F_{x}$). Hysteresis is calculated using the loading and unloading cycles shown in the enlarged view of Figure 14 and Figure 15. The sensor has a maximum hysteresis error of 0.45% ($T_{x}$). Resolution is calculated from the signal-to-noise ratio (SNR) in the no-load range, with results ranging from 0.55 to 0.94 for forces and 0.006 to 0.011 for torques.

To validate the performance of the proposed sensor, we compared its specifications with commercial sensors and research prototype sensors, as summarized in Table 4. The commercial sensor [31] provides a rate capacity of 100 N, and it has a diameter of 30 mm and height of 23.2 mm. In contrast, proposed sensor achieves the same 100 N capacity with a reduced height of 12 mm. Furthermore, compared to miniature sensors such as [3,18], which are typically limited to a range of 10 N, the proposed sensor offers ten times rate capacity for making a suitable robotic gripper. The accuracy (<0.96%) also surpasses that of the commercial miniature sensor [32] (approx. 5%).

## 5. Conclusions

This paper introduces a compact six-axis force/torque (F/T) sensor based on a redundant capacitive sensing architecture (RCSA) to address the sensitivity degradation and performance limitations commonly encountered in miniaturized F/T sensor designs. Unlike conventional designs that rely on mechanical decoupling, the proposed sensor employs a compliant structure in which coupled deformations naturally occur and are detected through redundant capacitive sensing. By allowing multiple electrodes to respond simultaneously to applied loads, the sensing architecture maintains sensitivity and signal stability. Mechanical modeling and finite element analysis showed that the proposed compliant structure produces independent displacement patterns that remain distinguishable for all six force and torque components. As a result, the applied multi-axis loads can be resolved without requiring direction-specific electrode layouts or a mechanically separated sensing arrangement. In addition, the electrode gap is defined passively by fabrication tolerances, reducing alignment complexity and improving repeatability in compact sensor manufacturing. The fabricated prototype, with overall dimensions of 20mm×12mm, is experimentally evaluated under the rated measurement range. When a neural network-based calibration method is used, it achieves high linearity and low cross-axis error across all axes, showing that the proposed sensing architecture effectively provides well-decoupled and axis-consistent multi-axis F/T measurements. These results suggest that redundant capacitive sensing combined with a compliant structure provides a practical alternative to mechanically decoupled architectures for miniaturized six-axis F/T sensors. The proposed sensor is therefore suitable for robotic end-effectors, grippers, and manipulation systems that require a compact form factor, reliable multi-axis force and torque feedback.

## Figures and Tables

**Table 1:** Capacitance changes of four sensing electrodes (↑: Cap. increase), (↓: Cap. decrease).

Case	$C_{1}$	$C_{2}$	$C_{3}$	$C_{4}$	$C_{5}$	$C_{6}$	$C_{7}$	$C_{8}$	$C_{9}$	$C_{10}$	$C_{11}$	$C_{12}$	$C_{13}$	$C_{14}$	$C_{15}$	$C_{16}$
$F_{x}$	↓	↓	↑	↑	↓	↓	↓	↓	↑	↑	↓	↓	↑	↑	↑	↑
$F_{z}$	↓	↑	↓	↑	↓	↑	↓	↑	↓	↑	↓	↑	↓	↑	↓	↑
$T_{x}$	↓	↑	↓	↑	↓	↑	↑	↓	↑	↓	↑	↓	↑	↓	↓	↑
$T_{z}$	↓	↓	↑	↑	↓	↓	↑	↑	↓	↓	↑	↑	↓	↓	↑	↑

**Table 2:** Specifications of the developed six-axis F/T sensor.

Quantity	Value	Unit
Force range $\left(F_{x},F_{y},F_{z}\right)$	±100	N
Torque range $\left(T_{x},T_{y},T_{z}\right)$	±0.5	Nm
Resolution of forces	0.80,0.55,0.94	N
Resolution of torques	0.011,0.010,0.006	Nm
Dimension	20 × 12	mm (*D* × *H*)
Sampling rate	300	Hz

**Table 3:** Performance results of the developed six-axis F/T sensor.

	Accuracy	Linearity	Crosstalk	Hysteresis	Resolution
$F_{x}$	0.18	99.10	1.70	0.25	0.80
$F_{y}$	0.04	99.25	1.02	0.06	0.55
$F_{z}$	0.96	99.01	1.19	0.43	0.94
$T_{x}$	0.36	98.22	1.02	0.45	0.011
$T_{y}$	0.54	98.20	0.98	0.32	0.010
$T_{z}$	0.14	99.11	1.15	0.28	0.006

The above performance indicators are calculated based on the total output (FSO).

**Table 4:** Comparison of the proposed sensor with the other sensors.

Sensor/Reference	Sensing Principle	Size (D×H)	Force Range	Torque Range	Meas. Error
		(mm)	($F_{z}$, N)	($T_{z}$, Nm)	(Accuracy)
MiniONE [31]	Strain Gauge	30×23.2	±100	±1.5	<2.0%
MMS101 [32]	Piezo-resistive	9.6×9.0	±40	±0.4	<5.0%
Li et al. [3]	FBG	10 × N/A	10	0.1	<5.34%
CoinFT [18]	Capacitive	20×2.0	15	0.08	<1.1%
Proposed Sensor	Capacitive	20×12	±100	±0.5	<0.96%

## Data Availability

The data presented in this study are available on request from the corresponding author.
